# Genetic Structure of *Lutzomyia* *longipalpis* Populations in Mato Grosso Do Sul, Brazil, Based on Microsatellite Markers

**DOI:** 10.1371/journal.pone.0074268

**Published:** 2013-09-16

**Authors:** Mirella F. C. Santos, Paulo E. M. Ribolla, Diego P. Alonso, José D. Andrade-Filho, Aline E. Casaril, Alda M. T. Ferreira, Carlos E. S. Fernandes, Reginaldo P. Brazil, Alessandra G. Oliveira

**Affiliations:** 1 Programa de Pós-Graduação em Doenças Infecciosas e Parasitárias, Faculdade de Medicina - FAMED, Universidade Federal de Mato Grosso do Sul, UFMS, Campo Grande, Mato Grosso do Sul, Brazil; 2 Departamento de Parasitologia, Universidade Estadual Paulista “Júlio de Mesquita Filho”, Unesp, Botucatu, São Paulo, Brazil; 3 Referencia Nacional e Internacional de Flebotomíneos, Centro de Pesquisas “René Rachou”, CCPQRR-FIOCRUZ, Belo Horizonte, Minas Gerais, Brazil; 4 Laboratório de Imunologia, Universidade Federal de Mato Grosso do Sul, UFMS, Campo Grande, Mato Grosso do Sul, Brazil; 5 Laboratório de Patologia, Universidade Federal de Mato Grosso do Sul, UFMS, Campo Grande, Mato Grosso do Sul, Brazil; 6 Laboratório de Doenças Parasitárias, Instituto Oswaldo Cruz, FIOCRUZ, Rio de Janeiro, Rio de Janeiro, Brazil; 7 Laboratório de Parasitologia, Universidade Federal de Mato Grosso do Sul, UFMS, Campo Grande, Mato Grosso do Sul, Brazil; University of Arkansas, United States of America

## Abstract

**Background:**

*Lutzomyia* *longipalpis* (Diptera: Psychodidae) is the major vector of *Leishmania* (*Leishmania*) *infantum* and thus plays a crucial role in the epidemiology of American visceral leishmaniasis (AVL). This vector is the best studied species of sand fly in the Neotropical region. Many studies claim that this vector is in fact a species complex; however there is still no consensus regarding the number of species that belong into this complex or the geographical distribution of sibling species. The aim of the present study was to analyze the genetic relationships within *Lu. longipalpis* populations in the state of Mato Grosso do Sul (MS), Brazil.

**Methodology/Principal Findings:**

We collected 30 *Lu. longipalpis* (15 females and 15 males) from five localities (Campo Grande, Três Lagoas, Aquidauana, Miranda and Bonito) and 30 *Lu. Cruzi* from Corumbá, totaling 180 sandflies from MS, and 30 *Lu. longipalpis* from Estrela de Alagoas, state of Alagoas (AL), Northeast Brazil. We show that eight previously described microsatellite loci were sufficient in distinguishing *Lu. longipalpis* from *Lu. Cruzi*, which is a closely related species, and in differentiating between *Lu. longipalpis* collected in MS versus Estrela de Alagoas. Analyses of the genotypes revealed introgression between sympatric *Lu. longipalpis* and *Lu. Cruzi.*

**Conclusions/Significance:**

Our findings support the hypothesis of cryptic species within the *Lu. longipalpis* complex. Furthermore, our data revealed introgression between *Lu. longipalpis* and *Lu. cruzi.* This phenomenon should be further investigated to determine the level and incidence of hybridization between these two species. We also demonstrated that microsatellite markers are a powerful tool for differentiating sand fly populations and species. The present study has elucidated the population structure of *Lu. longipalpis* in MS and, by extension, the Neotropical *Lu. longipalpis* complex itself.

## Introduction

Leishmaniases are diseases caused by various species of protozoa of the order Kinetoplastida, family Trypanosomatidae and genus *Leishmania* that affect humans and various wild and domestic animals throughout the world. The disease may present different clinical forms, depending on the species of *Leishmania* involved in the infection and the relationship between the parasite and its host [1,2]. Visceral leishmaniasis is an anthropozoonosis characterized by chronic evolution and systemic involvement, which if untreated can result in death. In the Americas, *Leishmania* (L.) *infantum* is the etiological agent of the disease and Brazil accounts for over 90% of the cases on the continent [3-6].

American visceral Leishmaniasis (AVL) is widely distributed in the state of Mato Grosso do Sul (MS), where it has been reported in several counties [7]. In 2012, 245 cases of AVL were diagnosed throughout 28 localities in MS. Of the total cases in that year, 172 (70.2%) occurred in Campo Grande, followed by Rio Verde de Mato Grosso with 24 cases (9.8%), Aquidauana with 7 cases (2.9%) and Anastácio and Coxim with 6 cases each (2.4%) [8].

**Figure 1:**
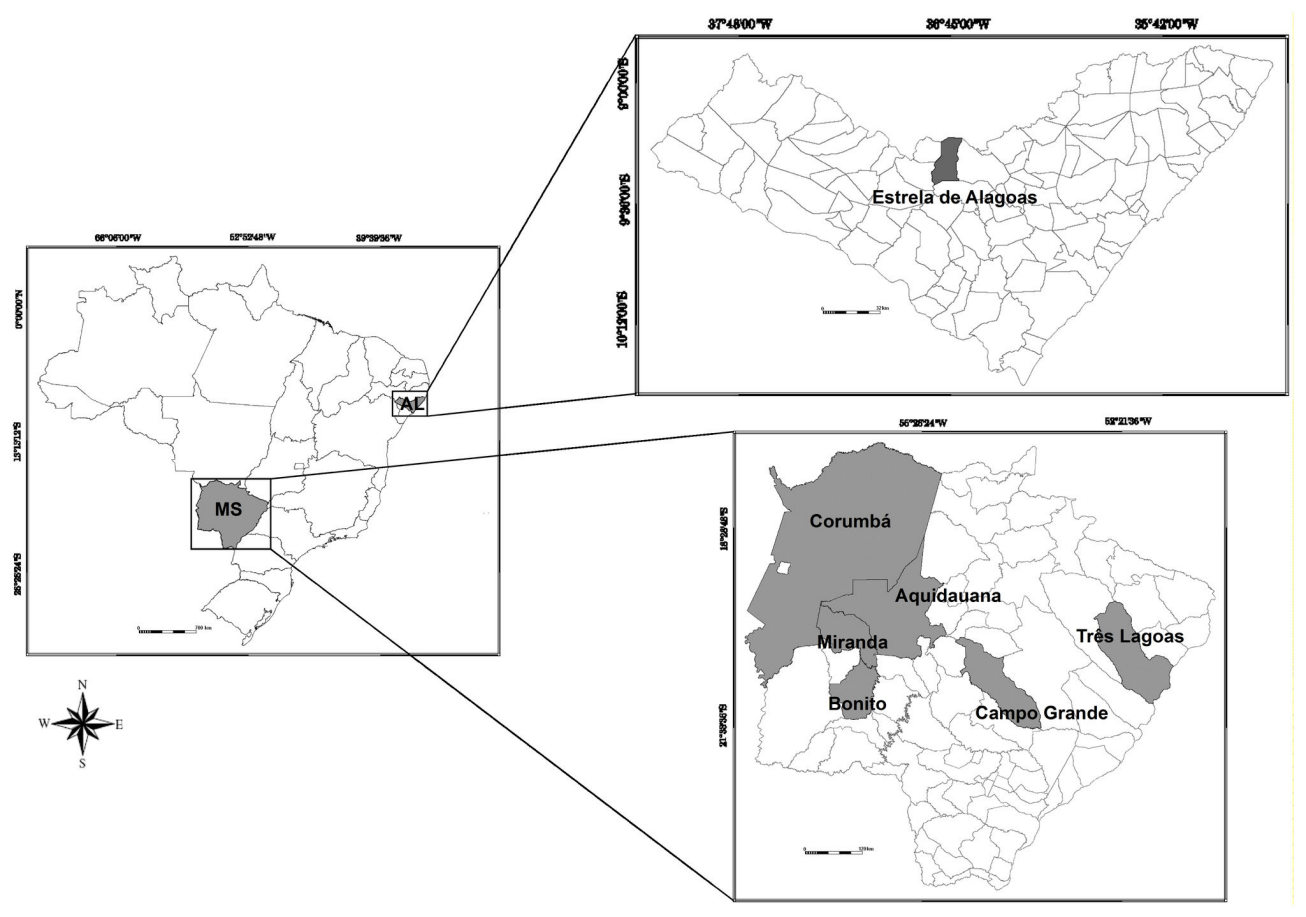
Map of Brazil, with emphasis on the states of Mato Grosso do Sul (MS) and Alagoas (AL), where the specimens of sandflies were collected. Map of Brazil demonstrating the distances of approximately 2.300 km between the states of Mato Grosso do Sul and Alagoas. The map also displays the localities where the specimens were collected in MS and in AL.

Leishmaniases are transmitted by an insect vector in the family Psychodidae. These vectors are distributed throughout the world, and in the Americas there are over 450 known species of sandflies. Approximately 60 of these species are involved in *Leishmania* transmission [9,10]. In Brazil, the main vector of AVL is *Lu. longipalpis*, except in Corumbá (MS) where *Lu. longipalpis* is absent. *Lu. cruzi* has been identified as a potential vector based on the collection of naturally infected flies [11,12].

Mangabeira [13] was one of the first to seriously discuss the taxonomic status of *Lu. longipalpis* in Brazil. He observed that males collected in the states of Ceará and Pará differed in the number of spots present on abdominal tergites. Namely, males collected in Ceará had a single pair of pale spots on tergite 4 (1S males), while males captured in Pará had an additional pair of spots on tergite 3 (2S males). This morphological variability was also noticed and investigated by Ward et al. [14] who performed crosses involving populations originated from Ceará (Morada Nova) and Minas Gerais (Lapinha). They demonstrated reproductive isolation between individuals 1S and 2S, further supporting the hypothesis of a "*longipalpis*" species complex. Since the emergence of this hypothesis and the discovery of cryptic species of *Lu. longipalpis*, a series of studies have aimed to clarify the taxonomic status of this species [15-26].

Advances in genetic and molecular technologies have provided the tools needed for the elucidation of the biosystematics, population dynamics and phylogenetic relationships among the various species of sandflies. Numerous molecular markers are currently available for entomological analysis and microsatellite loci have been identified and employed as markers in genetic and ecological population studies, including investigations in dipterans vectors of infectious diseases, such as *Anopheles* *darlingi* , a malaria vector [27,28] and *Aedes aegypti*, the vector for yellow fever and dengue fever viruses [29].

Microsatellites are simple sequence repeats arranged in tandem (SSR) of one to six nucleotides that occur throughout eukaryotic genomes. The number of repeats at a microsatellite locus may vary continuously between individuals of a species and are used as co-dominant markers [30]. To date, there is no data available on the sibling species that constitute the *Lu. longipalpis* complex in Mato Grosso do Sul, or information about the interrelationships between taxonomic species of sandflies found there. The specific aim of the present study was to analyze the genetic relationships, based on microsatellite loci, within and between *Lu. longipalpis* populations in the state of Mato Grosso do Sul, using a population from Northeast Brazil as an outlying group, and compare it with a population of *Lu. cruzi*.

## Methods

### Ethics Statement

For insect collections we obtained a permanent license for collecting and transporting zoological material N° 25592-1 on behalf of Dr. Alessandra Gutierrez de Oliveira, issued by the System of Authorization and Information on Biodiversity of the Brazilian Institute of Environment and Renewable Natural Resources (Sisbio/IBAMA). The collections were performed at private residences, whose owners personally granted permission to enter their backyards to capture the sandflies. All of these residences were located in urban areas and no endangered or protected species were collected in this study.

### Sandfly Populations

The phlebotomines were captured using modified CDC light traps from 6 p.m. to 6 a.m. and motorized aspirators at dusk and during the early night hours. We collected *Lu. cruzi* from Corumbá and *Lu. longipalpis* from five other localities in MS: Miranda, Aquidauana, Bonito, Campo Grande and Três Lagoas. We also included *Lu. longipalpis* specimens from Estrela de Alagoas – AL for use as an outgroup in genetic analyses (Figure 1). All sandflies were identified based on morphological characteristics of the genitalia, head, and thorax, as described by Galati [10]. Species confirmation was only performed on male individuals because females of the two species are indistinguishable [31]. We observed the expected pattern of tergite spots in *Lu. longipalpis* and *Lu. cruzi* individuals (Table 1). All of the sandflies from Corumbá, Miranda, Aquidauana, Bonito and Três Lagoas had 1S. In Campo Grande there were eleven 1S individuals and four 2S individuals. In Estrela de Alagoas we collected eight individuals with 1S and seven with 2S.

**Table 1:** Collection sites and number of tergite spots in *Lutzomyia* *longipalpis* and *Lutzomyia* *cruzi* from Mato Grosso do Sul and Alagoas.

**Species**	**Tergite spot pattern**	**Localities, State**	**Coordinates - Latitude/Longitude**
***Lu.longipalpis***	1S and 2S	Campo Grande, MS	20°26'34″ S/54°38'47″ W
	1S	Aquidauana, MS	20°28'16″ S/55°47'14″ W
	1S	Bonito, MS	21°07'16″ S/56°28'55″ W
	1S	Miranda, MS	20°14'26″ S/56°22'42″ W
	1S	Três Lagoas, MS	20°45'04″ S/51°40'42″ W
	1S and 2S	Estrela de Alagoas, AL	09°23'25″ S/36°45'36″ W
***Lu.cruzi***	1S	Corumbá, MS	19° 00' 33″ S/57°39'12″ W

1S, one pair of tergite spot. 2S, two pair of tergite spots. MS, Mato Grosso do Sul. AL, Alagoas

### Extraction of DNA

After morphological identification, the insects (15 males and 15 females) from each study locality were preserved in 70% alcohol and subsequently crushed using a plastic pestle and portable mixer in 1.5 mL tubes containing 300 µL of 5% Chelex^®^ Molecular Biology Grade Resin (Bio-Rad Laboratories, Hercules, USA) according to the manufacturer’s recommendations. The solution was mixed by vortex for 15s and subsequently subjected to 20 s of centrifugation at 11,000 g. Next, the solution was placed in a water bath at 80°C for 30 min, after which the procedure was repeated. The supernatant was removed, transferred to another sterile Eppendorf tube, and frozen at -20°C.

### Microsatellite PCR amplification

To amplify potentially polymorphic regions in genomic DNA, PCR was performed using eight primers described by Watts et al. [32] as listed in Table 2. Twenty five µL of PCR reactions were prepared as follows: 12.5 µL of Gotaq Colorless Master Mix (Madison, WI, USA), 1.0 µL of each oligonucleotide (10 pmol/µL), and 5.0 µL of DNA. Due to the difference in annealing temperature of some oligonucleotides, we standardized two protocols. The reactions were placed in a thermal cycler (Biometra T Gradient, USA) for 5 cycles of denaturation at 94 °C for 15 s, annealing 50/53 °C for 30 s, elongation at 68 °C for 30 s, followed by 25 cycles of denaturation at 94 °C for 15 s, annealing at 50/53 °C for 30 s and elongation at 70 °C for 30 s, and a final extension of 70 °C for 5 min. Once the optimal conditions for PCR were established, reverse primers were labeled with fluorochromes (HEX or FAM). The PCR products obtained with tagged primers were subsequently subjected to fragment analysis in an automated sequencer.

**Figure 2:**
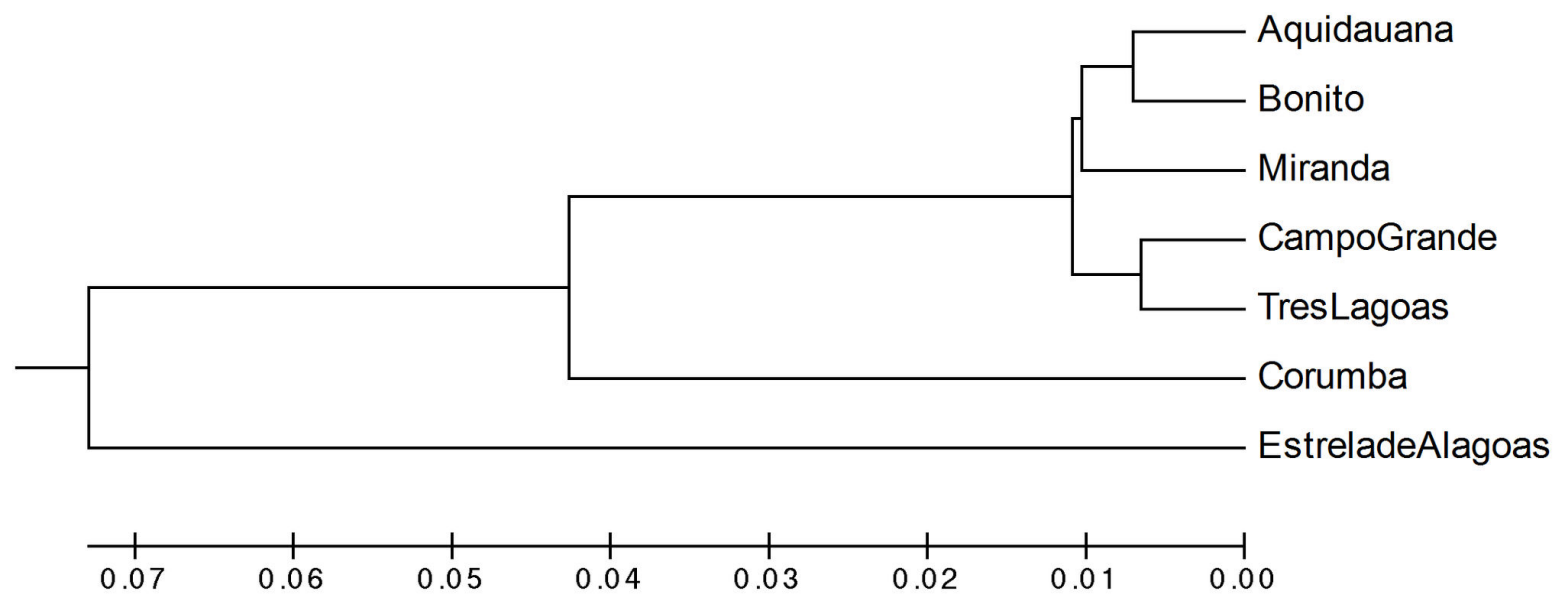
The genetic distance between *Lu. cruzi* and *Lu. longipalpis* by locality UPGMA dendrogram presenting the genetic distance between *Lu. cruzi* (Corumbá) and *Lu. longipalpis* (all of the others) by locality. The dendrogram is based on all of the pair-wise F*st* estimates and indicates a genetic difference between the two groups in MS and between these groups and the outgroup.

**Table 2:** Oligonucleotide panel according to Watts *et al.* [30].

**Locus**	**Primer sequence (5’–3’**)	**Repeat motif**	**GenBank access no.**
LIST6001	AAAGGGTGCGAAGTTATTGC	(CA)17... (GA)4	AF411613
	GGGTGGGTTGGACATTCTAC		
LIST6005	CCCTCCTTCTTACAACTCACC	(TG)9	AF411616
	ACCTTTATCGGCCCAGGTAG		
LIST6006	CCCATTCTGTGTGTTGTCGT	(AC)15	AF411617
	TGCACATTTGAGTGGTTTGTG		
LIST6007	GCTCCCATTTAATCTCCATAC	(AC)21	AF411618
	CGCCAAGACAAACATTTTCC		
LIST6012	AAATGGGTGGGTCAAGAGTG	(GT)6... (GT)	AF411620
	GTCGCACGGTCTTATGGAAA		
LIST6014	TTTCGTCTTCTTCAGGCAGTC	(CA)3... (CA)... (CA)5	AF411621
	AAAGTCACCACCCGAGAAAG		
LIST6025	TTTCGACACCACGTTATGGA	(TG)3... (TG)6	AF461123
	ATCCTCCGCCTACGCTTTAC		
LIST6029	AACACGCTGGGATTGAA	(TG)8(TC)2(TG)	AF461125
	CATCTGAAGTGAATGAGGAAGG		

### Microsatellite data analysis

The PCR products were separated on an automated sequencer (ABI 3700, Applied Biosystems, USA), and their sizes and allele peaks were analyzed using the Peak Scanner (Applied Biosystems, USA) software. The genetic and molecular data were analyzed with the MICROSAT program [33] and MEGA4 software [34]. Structure 2.3.3 [35] was used to determine the population structure based on multilocus genotypic data. Cluster analysis was performed using prior information about the geographic collection points and employing the option of admixture, i.e., assuming a degree of ancestry within individuals. Twenty independent replicate runs, each with 50,000 iterations after a burn-in of 100.000 iterations, were performed for the Δk statistic to infer the optimal number of clusters (k).

**Figure 3:**
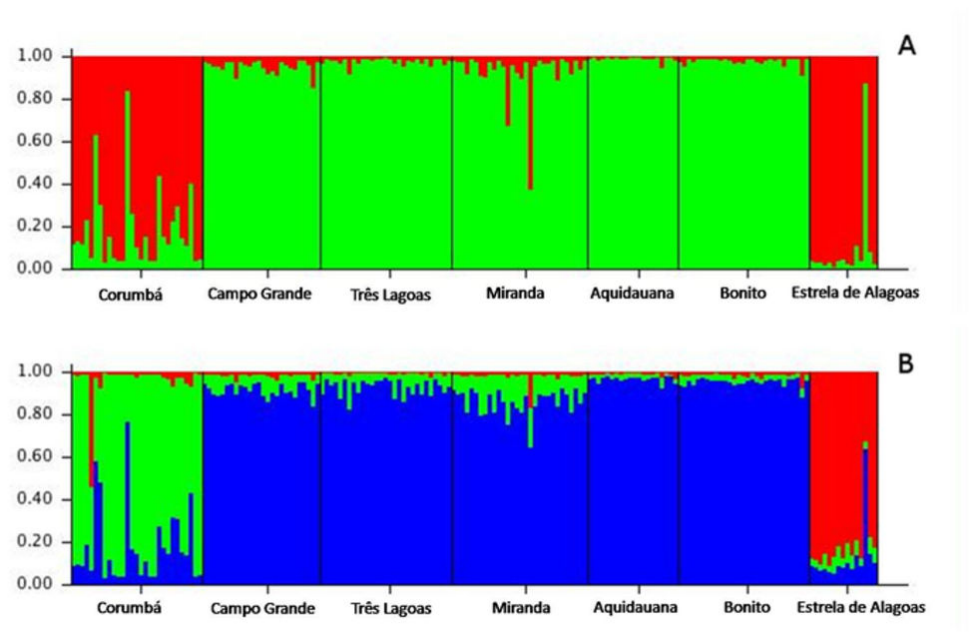
Studied populations inferred from STRUCTURE analysis based on profiles of 8 microsatellites. (A) The numbers under each square stand for 1: Corumbá, 2: Campo Grande, 3: Três Lagoas, 4: Miranda, 5: Aquidauana, 6: Bonito, 7: Estrela de Alagoas. Each color represents samples that have a similar genetic background. Using Δk, 2 populations were obtained. (B) In this picture, Ln (k) was used, and the results reveal 3 assigned populations.

## Results

### Descriptive analyses

Analysis of microsatellite fragments generated from 180 samples of phlebotomines from Mato Grosso do Sul, were performed to evaluate genetic similarity between the samples. The overall variation of allele sizes was transformed in a genetic distance matrix that was used to calculate a matrix of pairwise allele fixation indices (Fst). Significant differences were observed among all MS populations of *Lu. longipalpis* compared with the population of *Lu. cruzi* (Table 3). Fst values ranged from 0.71 (Miranda) to 0.101 (Bonito), with an even higher difference from the Estrela de Alagoas *Lu. longipalpis* population; the Fst ranged from 0.141 (Bonito) to 0.157 (Três Lagoas). The phenetic UPGMA tree based on all pairwise Fst estimates illustrated the differences between the two different vectors in MS (*Lu. longipalpis* grouped on the upper part of the tree but still separated from *Lu. cruzi*) and between those MS sandflies and the outgroup (Estrela de Alagoas – AL) at the base of the tree (Figure 2). Importantly, no genetic difference was found when the two tergite-spot phenotypes were assessed separately.

**Figure 4:**
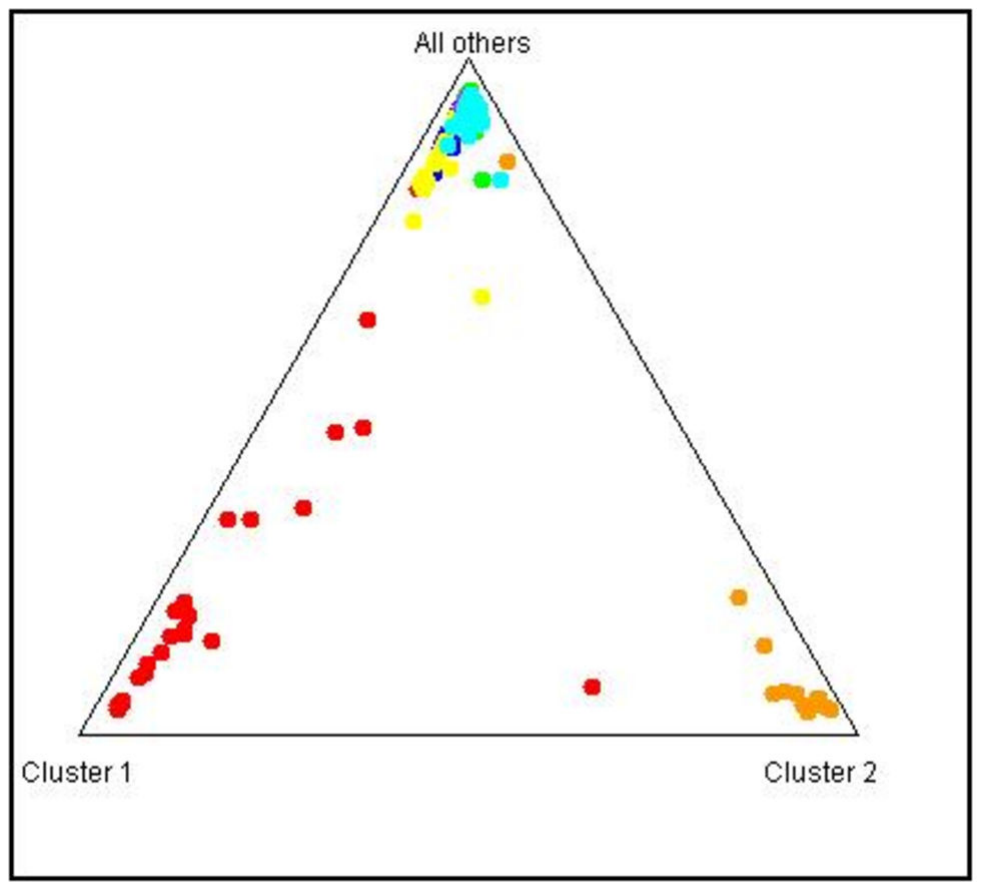
STRUCTURE Triangle plot showing the distribution by similarity. The dots represent each individual, and the colors represent the localities: Red: Corumbá, Orange, Estrela de Alagoas, Blue: Três Lagoas, Green: Campo Grande, Yellow: Miranda, Violet: Aquidauana, Light Blue: Bonito. The outgroup Estrela de Alagoas (in orange) is clearly distanced from the other populations. The population of Corumbá (in red) is also detached from the other populations but with a small distance compared with the outgroup.

**Table 3:** The standard fixation of alleles (Fst) of *Lu. longipalpis* and *Lu. cruzi* populations from Mato Grosso do Sul and Alagoas, Brazil.

	Corumbá	Campo Grande	Três Lagoas	Miranda	Aquidauana	Bonito	Estrela de Alagoas
Corumbá	0.000	-	-	-	-	-	-
Campo Grande	0.072*	0.000	-	-	-	-	-
Três Lagoas	0.085*	0.013	0.000	-	-	-	-
Miranda	0.071*	0.016	0.026*	0.000	-	-	-
Aquidauana	0.097*	0.016	0.021	0.017	0.000	-	-
Bonito	0.101*	0.022	0.029*	0.024*	0.014	0.000	-
Estrela de Alagoas	0.136*	0.143*	0.157*	0.144*	0.154*	0.141*	0.000

Significant Fst values are marked with * (significance level = 0.05)

### Population Structure Analyses

We obtained different results depending on the algorithm used in Structure [35] to estimate the best value for k. Using Δk, 2 genetic clusters (one red and one green) were found to outline the populations (Figure 3a), and the Corumbá and Estrela de Alagoas populations contained much more of the red cluster compared with the other MS populations. In contrast, when Ln (k) was used, 3 genetic clusters were produced (Figure 3b); in this figure the green cluster is almost exclusive for Corumbá. The same finding applied to the blue cluster, which is the major cluster for the other populations of MS, and the red cluster, which was found in a major proportion of the population of Estrela de Alagoas. Figure 4 presents the clustering between all of the populations. The population of Corumbá (in red) is clearly isolated from the other populations, including the outgroup of Estrela de Alagoas (in orange).

## Discussion

Based on these results, we conclude that *Lu. longipalpis* from MS and AL are diverging populations, consistent with the geographic distance of at least 2.300 Km between these populations compared with the distance observed for all MS populations (654 Km between Três Lagoas and Corumbá - Figure 1). Furthermore, analysis of allele frequency variation clearly revealed differences between *Lu. longipalpis* populations in MS and the *Lu. cruzi* population.

These findings were congruent with cluster phenetic reconstructions based on genetic distances (Figure 2) and, after applying a Bayesian model-based approach, three main vector populations were identified, corresponding to three separate locations: Corumbá, all other municipalities in MS, and Estrela de Alagoas (Figure 3 and Figure 4). Our data also suggest that *Lu. longipalpis* and *Lu. cruzi* in Mato Grosso do Sul are not fully reproductively isolated, probably due to the close proximity between these populations, and are two separate sibling species. In Mato Grosso do Sul, there is evidence of the sympatric occurrence of *Lu. longipalpis* and *Lu. cruzi* in Campo Grande and Bonito, despite the prevalence of *Lu. longipalpis* in both localities [36,41], although the catches have not demonstrated sympatry between these different vector populations, suggesting evidence of past introgression.

For insect disease vectors, gene flow between species may yield important epidemiological consequences, because genes controlling aspects of vectorial capacity might be transferred from one species to the other, perhaps changing the disease patterns [42,43]. Based on this rationale we infer that *Lu. cruzi* is part of *Lu. longipalpis* complex, and introgression may be occurring, which could lead to further species isolation. Possible morphological hybrids were revealed by the presence of the tuft of setae on gonocoxites that appeared to be a mixture of the two phenotypes as observed several times when analyzing *Lu. longipalpis* from those localities during routine work in our laboratory.

So far, populations of *Lu*. *Longipalpis* have been studied such as analysis of hybridization [05], sex pheromones [15,21,44], isozyme profile analysis [14,45], cytogenetic characters [37], male courtship songs [20,24,38,46], analysis of mitochondrial DNA [45] and microsatellite markers [39,40]. The results of several of these studies support the hypothesis of the existence of a *Lu*. *longipalpis* species complex [14,15,45], however, no consensus exists regarding the number and distribution of the different sibling species [23,45,47], although several researchers have demonstrated that, in Brazil, the sibling species differ in their male copulation songs, pheromones and molecular markers [22,25,26,36].

We demonstrate in our study compelling evidence of introgression between *Lu. longipalpis* and *Lu. cruzi*. This phenomenon should be fully investigated in future studies to determine which features are implicated in this process. This work presents significant advances in the understanding of the population structure of *Lu. longipalpis* in MS and, by extension, the neotropical *Lu. longipalpis* complex itself. We believe that assessing the genetic structure of vector populations may ultimately shed light on sand fly evolution regarding vector-parasite interactions, which might be an essential aspect of the eco-epidemiology of American Visceral Leishmaniasis.
